# Preliminary Study on Host Use and Phylogenetic Analysis of *Corethrella nippon* in Taiwan

**DOI:** 10.1002/ece3.72405

**Published:** 2025-10-30

**Authors:** Woo Jun Bang, Jh Yu You, Yoonhyuk Bae, Ming‐Feng Chuang, Seunggwan Shin

**Affiliations:** ^1^ School of Biological Sciences Seoul National University Seoul Republic of Korea; ^2^ Comparative Medicine Disease Research Center Seoul National University Seoul Republic of Korea; ^3^ Department of Life Sciences and Research Center for Global Change Biology National Chung Hsing University Taichung Taiwan; ^4^ Department of Agricultural Biotechnology Seoul National University Seoul South Korea; ^5^ Global Change Biology Research Center National Chung Hsing University Taichung Taiwan

**Keywords:** DNA barcodes, frog‐biting midges, frog‐biting mosquitoes, molecular phylogeny

## Abstract

This study investigated frog‐biting dipteran species using newly designed frog‐calling traps in Taiwan. The trap effectively collected specimens from both families, Culicidae and Corethrellidae, demonstrating its utility. Host preference analysis revealed that *Odorrana swinhoana* (Boulenger, 1903) and *Kurixalus eiffingeri* (Boettger, 1895) were most frequently associated with collected specimens of Corethrellidae. Additionally, the corethrellids were predominantly attracted to a sound frequency around 2200 to 2700 Hz. Then, DNA barcoding was also conducted on the four collected species of Culicidae: *Armigeres subalbatus* (Coquillett, 1898), *Uranotaenia nivipleura* Leicester, 1908, *Ur*. *macferlanei* Edwards, 1914, and *Mimomyia luzonensis* (Ludlow, 1905), and the mitochondrial genome of *Corethrella nippon* Miyagi 1980 was first sequenced and annotated. Mitogenome‐based phylogenetic analysis confirmed that *C. nippon* formed a clade with *Corethrella condita* Borkent, 2008. In our analysis, family Corethrellidae clustered with Culicidae; however, the inter‐family phylogenetic relationships within Culicoidea appeared paraphyletic, particularly concerning family Chaoboridae. Future studies should explore a greater variety of frog species across more diverse regions and use other genomic datasets beyond the mitogenome to infer a more robust deep topology at the superfamily level and further broaden our understanding of host preference.

## Graphical abstract

This study evaluated a new frog‐calling trap in Taiwan that successfully collected the species of Culicidae and Corethrellidae and identified host preferences, with Corethrellidae most frequently associated with *Odorrana swinhoana* and *Kurixalus eiffingeri* (mostly attracted to ~2022 Hz calls). We also DNA‐barcoded four collected culicid species and sequenced the complete mitochondrial genome of *Corethrella nippon*, whose phylogeny grouped it with *C. condita*. Mitogenome‐based analysis revealed a paraphyletic Culicoidea, grouped with Corethrellidae within Culicidae.

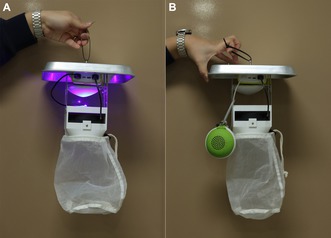

## 1 Introduction

Family Corethrellidae (Diptera), whose common name is frog‐biting midges, belongs to the superfamily Culicoidea and is a phylogenetic lineage closely related to Culicidae (Borkent 2014). This group is known for its unique evolutionary trait of hematophagy, exclusively feeding on the blood of amphibians, particularly anurans, and is observed to be attracted to male frog calls (McKeever 1977; Bernal and de Silva 2015; Zhao et al. 2022). This family has been recorded with over 100 species globally and is distributed across subtropical and tropical regions (Stone 1968; Borkent 2008). In East Asia, corethrellids are commonly found in the Ryukyu Archipelago of Japan, southern China, and have been recorded once in North Korea (Borkent 2014). In Taiwan, the only species, *Corethrella nippon* Miyagi 1980, was first reported in 1998, but little subsequent research has been conducted on this family (Lien et al. 1998). Moreover, although extensive research on this group has been conducted in North and South America, taxonomic and ecological studies on this species remain insufficient in Asia (Borkent 2008). Even then, the research has been limited to a few studies on the Ryukyu Archipelago of Japan, highlighting the greater need for foundational research on this family (Toma et al. 2005, 2019). In addition, a certain mosquito genus, *Uranotaenia* Lynch Arribálzaga, 1891, is known to feed on the blood of frogs, similar to frog‐biting midges (Borkent and Belton 2006). A total of six species have been recorded in Taiwan: *Ur*. *maxima* Leicester, 1908, *Ur*. *nivipleura* Leicester, 1908, *Ur*. *novobscura* Barraud, 1934, *Ur*. *yaeyamana* Tanaka, Mizusawa & Saugstad, 1975, *Ur*. *annandalei* Barraud, 1926, *Ur*. *macferlanei* Edwards, 1914 Lien 2004. However, most *Uranotaenia* species have been collected as larvae, with no research investigating whether adult mosquitoes are directly attracted to frog calls. Therefore, this study aims to test whether adult Corethrellidae and Culicidae (especially the genus *Uranotaenia*) in Taiwan respond to frog calls.

In addition to the ecological studies, molecular analyses of the collected species are essential for accurate species identification. To achieve this, mtCOI‐based genetic analyses were conducted on both Culicidae and Corethrellidae specimens to obtain DNA barcodes, aligning with the Gao et al. (2017) to improve the accuracy of species identification for future research.

Finally, we aim to investigate the mosquitoes and frog‐biting midges by using the calling sounds of frog species in Taiwan, drawing upon research conducted by Toma et al. (2005) and Toma et al. (2019) on Iriomote Island, Ryukyu Archipelago, Japan, based on the newly designed traps. Our objectives are threefold: first, to compare the number of collected specimens between different geographical characteristics: mountainous and valley regions; second, to identify which common frog calls attract the highest number of specimens; third, to present the molecular data (mtCOI sequences and mitochondrial genome) of the collected species for further studies.

## 2 Methods and Materials

### 2.1 Newly Designed Trap Using 3D Printer

**FIGURE 1:**
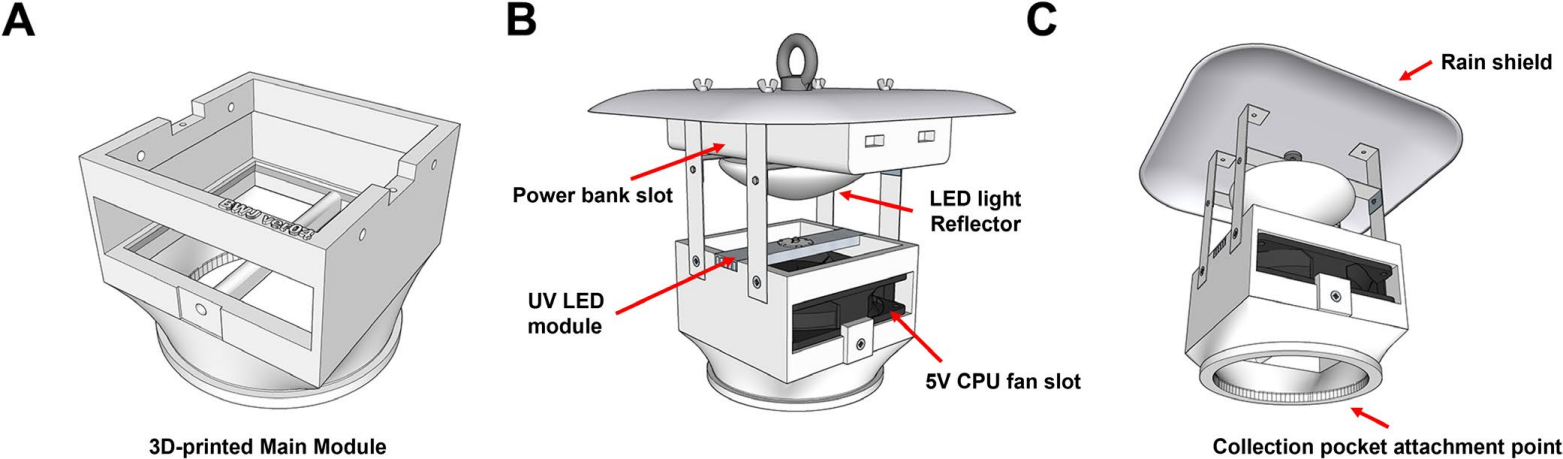
The schematic of the newly designed trap. (A) 3D‐printed main module; (B, C) Explanation of key components of the trap.

We used the three newly designed traps, which were developed for collecting mosquitoes and frog‐biting midges. The trap was developed based on the method described by Toma et al. (2005) and designed using the SketchUp Pro 2023 program (Figure 1). Based on the finalized schematic, the main body was constructed using a 3D printer (Model Ender‐3 V3 KE, Creality Corp., Shenzhen, China). Additional components, including aluminum strips, UV LEDs (3 W UV Purple LED 365 nm, Qingying Corp., Guangdong, China), LED heat sinks (Aluminum Alloy Heatsink, 150 × 20 × 6 mm, DYHF Corp., China), a step‐down power module (Mini360 DC‐DC HM Buck Converter, Tenstar Robot Corp., Shenzhen, China), and a 5 V cooling fan (Model DC9025, Gakaki Corp., Shenzhen, Guangdong, China) for insect collection, were integrated to provide grounding and auxiliary functions (Figure S1). All components were designed for portability, as well as easy assembly and disassembly.

The trap was designed to be powered by a power bank (Model PB2022ZM; 20,000 mAh Power Bank 2C, Xiaomi Corp., Beijing, China), enabling continuous operation for up to 40 h. A wireless speaker (Model A110MINI; EWA Corp., Zhumadian, Henan, China) positioned near the fan plays frog calls to effectively attract both frog‐biting midges and mosquitoes.

**FIGURE 2:**
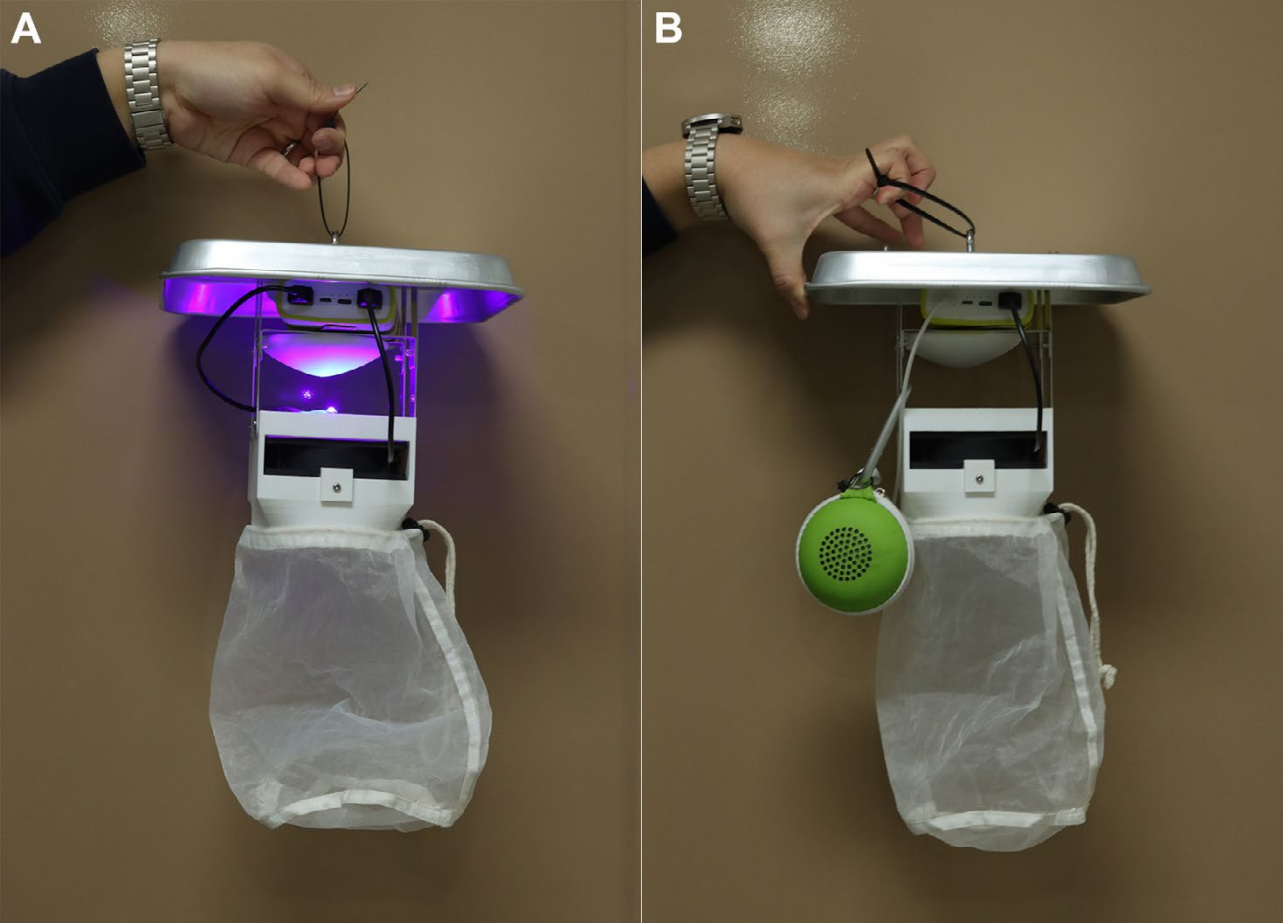
Two trap configurations. (A) Light trap version optimized for light‐attracted insects; (B) Frog‐biting flies trap version configured for frog sounds.

The trap was designed with two configurable versions to target specific insect specimens. In the first version, optimized for collecting frog‐biting midges and mosquitoes, the UV LED was excluded, and only the wireless speaker and fan were powered by the power bank (Figure 2A). In the second version, the wireless speaker was deactivated, and the UV LED, along with the fan, was powered by the power bank (Figure 2B).

In summary, compared to the conventional CDC trap or other known miniature traps, it is highly portable due to its small size (230 × 166 mm) and is easy to disassemble. Additionally, for collecting frog‐biting flies, the wireless speaker and fan were activated, whereas, for light‐attracted insects, the UV LED replaced the wireless speaker, with the fan remaining operational.

### 2.2 Field Survey and Identification

**FIGURE 3:**
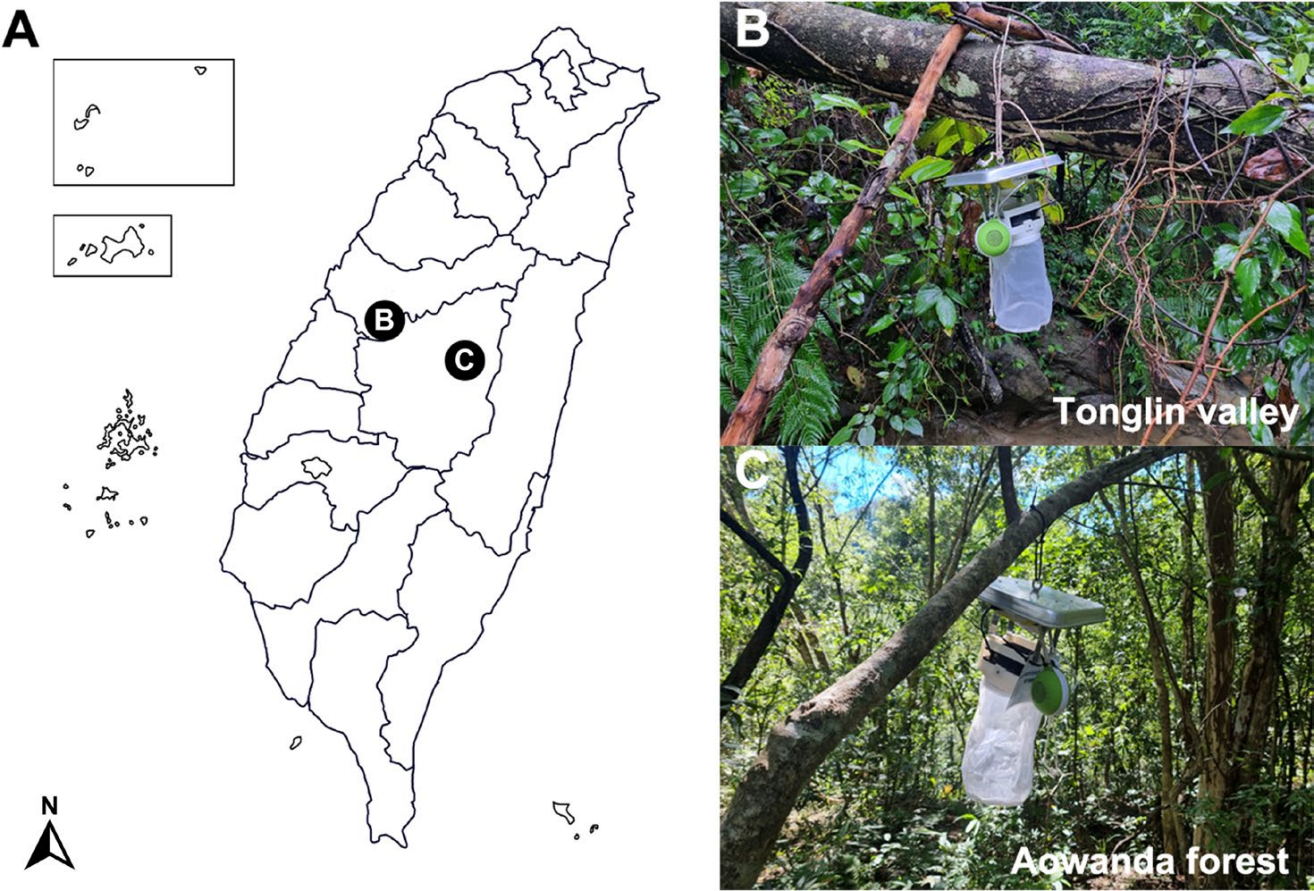
(A) Collection sites in Taiwan, (B) Tonglin valley, (C) Aowanda forest.

Field surveys were conducted in the mountainous Aowanda National Forest Recreational Area, Nantou (23°58′27.0″ N 121°07′51.5″ E) from July, and in the valley region of Tonglin, Wufeng District, Taichung (24°02′52.3″ N 120°47′05.3″ E) from July and September (Figure 3). Finally, the three traps (version of Figure 2B) with frog calls were activated from 5:00 p.m. to 10:00 a.m. the following day, with one serving as the control. Collected specimens were identified to species following Miyagi (1980), Lien et al. (1998), and Lien (2004); other collected Diptera specimens were sorted and identified at the family level. Finally, the species composition and number of collected specimens were analyzed and compared to frog calls and geographical characteristics, respectively.

### 2.3 Selection of Frog Calls

**FIGURE 4:**
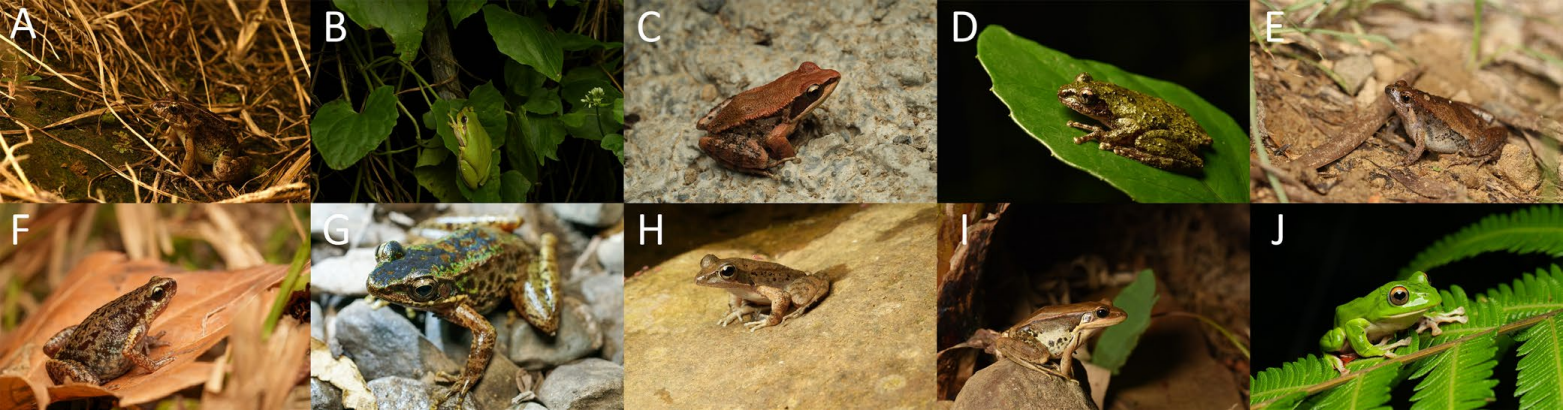
The frog species used in host preferences analysis. (A) *Fejervarya limnocharis* (B) *Hyla chinensis* (C) *Hylarana latouchii* (D) *Kurixalus eiffingeri* (E) *Microhyla fissipes* (F) *Micryletta steinegeri* (G) *Odorrana swinhoana* (H) *Rana sauteri* (I) *Sylvirana guentheri* (J) *Zhangixalus moltrechti* .

We employed eight different frog calls for the survey (mixed frog calls): *Hyla chinensis* Günther, 1858; *Hylarana latouchii* (Boulenger, 1899); *Sylvirana guentheri* (Boulenger, 1882), *Micryletta steinegeri* (Boulenger, 1909), *Kurixalus eiffingeri* (Boettger, 1895), *Fejervarya limnocharis* (Gravenhorst, 1829); *Zhangixalus moltrechti* (Boulenger, 1908), and *Odorrana swinhoana* (Boulenger, 1903). The species of frog calls were selected according to the local frog fauna. Additionally, we investigated to ensure the trap is working to forage mosquitoes and frog‐biting midges at the same location in Tonglin, including the eight frog species used for the mixed frog call and separate frog calls, along with the commonly found *Rana sauteri* (Boulenger, 1909) and *Microhyla fissipes* (Boulenger, 1884), resulting in a total of ten frog species whose calls were analyzed to assess host preferences (Figure 4).

**FIGURE 5:**
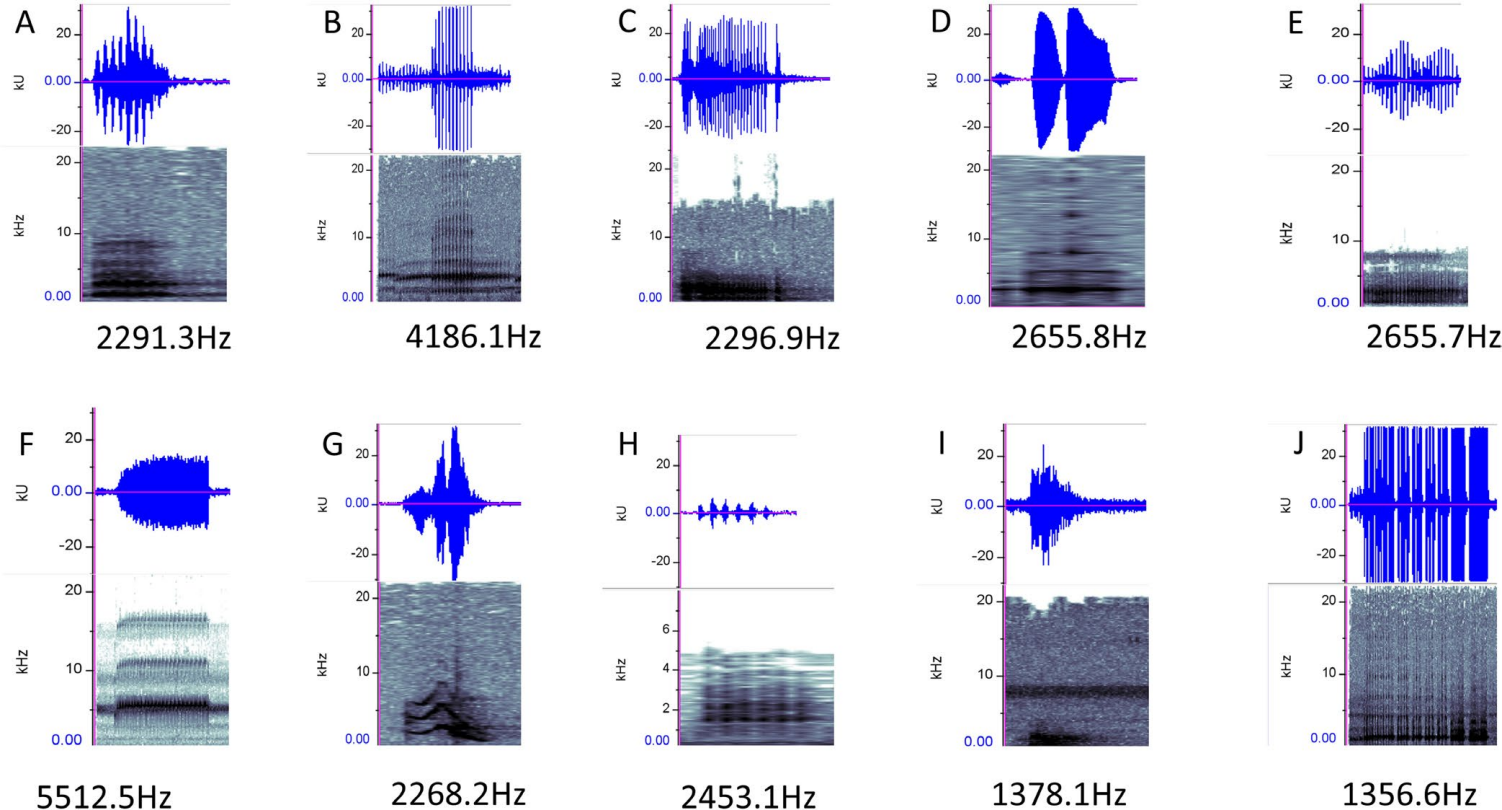
The waveform (upper panel) and spectrogram (lower panel) of the sound of each frog call used in the experiment; the numbers represent the average dominant frequency of the frog calls we used. (A) *Fejervarya limnocharis* (B) *Hyla chinensis* (C) *Hylarana latouchii* (D) *Kurixalus eiffingeri* (E) *Microhyla fissipes* (F) *Micryletta steineger* (G) *Odorrana swinhoana* (H) *Rana sauteri* (I) *Sylvirana guentheri* (J) *Zhangixalus moltrechti*.

The trap was conducted in the valley region of Tonglin, Wufeng District, Taichung (24°02′52.3″ N 120°47′05.3″ E). Three traps were set on trees near the tributary of the Beikeng River and activated daily from 5:00 p.m. to 10:00 a.m. the following day. To avoid interference from the frog calls emitted by other traps, each trap was placed at least 100 m apart. The dominant frequencies of the frog calls used in the study ranged from 1356.6 Hz to 5512.5 Hz (Figure 5). Each species' call was tested once, and all insects trapped during that period were collected after counting and identifying the specimens.

### 2.4 Molecular Data Acquisition

The genomic DNA (gDNA) was extracted from the whole body or one grounded leg, using an OmniPrep for Tissue Kit (Cat. #786–395; G‐Biosciences, USA). A partial region of the mitochondrial cytochrome *c* oxidase I (mtCOI) was amplified by polymerase chain reaction (PCR) as the following procedures: 94°C for 5 min for denaturation; 35 cycles of 94°C for 30 s, 50°C for 40 s, and 72°C for 40 s; with a final extension for 5 min at 72°C, with a reaction mixture containing a total volume of 25 μL: 1× PCR buffer, 0.4 μM each primer LCO 1490 [5′‐GGTCAACAAATCATAAAGATATTGG‐3′ (forward)] and HCO 2198 [5′‐TAAACTTCAGGGTGACCAAAAAATCA‐3′ (reverse)] (Folmer et al. 1994), 1.5 mM MgCl_2_, 0.2 mM each dNTP, 0.5 units of Taq DNA polymerase (R001AM; Takara Bio, Kusatsu, Shiga, Japan), and ~1.0 ng of extracted DNA. Each PCR product was sequenced in both directions by Bionics Corp. (Seoul, Republic of Korea). Then raw sequences were assembled and trimmed using Geneious Prime 2024.0.7 and finally deposited to GenBank as the following accessions: PV576006, PV576008, PV576009, PV576069.

### 2.5 Mitochondrial Genome Sequencing, Assembly and Annotation

About *Corethrella nippon*, ~150 ng of sample DNA was used for library preparation for short read sequencing for further mitochondrial genome extraction. The DNA was fragmented to a size of 300–400 bp using Q800R3 Sonicator Chromatin and DNA Shearing System (Qsonica, USA). A short‐insert library was then constructed using the NEBNext Ultra II DNA Library Prep Kit for Illumina (Cat. E7645L; New England Biolabs, USA) according to the manufacturer's instructions. Sequencing was conducted on an Illumina NovaSeq 6000 platform, producing ~5 Gb (Marcrogen Corp., Republic of Korea).

The raw data was processed using Trimmomatic v0.39 (Bolger et al. 2014) for adapter trimming, and the mitochondrial reads were subsequently used to assemble the mitochondrial genomes by using MitoFinder (Allio et al. 2020). Due to the absence of complete mitochondrial genome data for the family Corethrellidae in GenBank, *Dixella aestivalis* (Meigen, 1818) (GenBank accession: NC_029354.1) from the closely related family Dixidae was used as a reference. Manual curation was conducted using Geneious Prime 2024.0.7, and the finalized .gb file was uploaded to Proksee (Grant et al. 2023) for visualizing the mitochondrial circular map, where GC content and GC skew were also calculated as part of the analysis as follows: AT skew = (A% − T%)/(A% + T%)/GC skew = (G% − C%)/(G% + C%). The final assembled mitochondrial genome was deposited in GenBank with the following accession: PV605830.

### 2.6 Data Processing and Phylogenetic Analysis

The preliminary phylogenetic analysis was also conducted to determine the phylogenetic position of *C. nippon* with other species of Culicoidea (Diptera). A total of 12 mitochondrial genomes were analyzed for this study. This dataset includes two genomes from Dixidae (*Dixella aestivalis*—NC_029354.1 and *Dixella* sp. ZK‐2014—KM245574.1), three from Chaoboridae (*Chaoborus* sp. ZK‐2019—MK281356.1, *Chaoborus trivittatus* extracted from the SRA repository: SRR1738278, and *Mochlonyx cinctipes* extracted from the SRA repository: SRR1738194), two from Corethrellidae (*Corethrella condita*—MK281357.1 and a newly sequenced mitochondrial genome of *Corethrella nippon*), and four from Culicidae (*Anopheles sinensis*—OK458560.1, *Aedes albopictus* —AY072044.1, *Culex tritaeniorhynchus*—KT851544.1, and *Uranotaenia geometrica*—MK575491.1). Additionally, one outgroup species, *Culicoides brevitarsis* (NC_085211.1), was included.

All sequences were aligned using MAFFT v7.475 software, then manually trimmed in Geneious Prime 2024.0.7. The concatenated alignment matrix of 13 protein‐coding genes (PCG) was assembled using FASconCAT‐G v1.02 (Kück and Longo 2014), and a maximum likelihood tree was constructed with IQ‐TREE v2.1.2 with the ultrafast bootstrap method and 1000 replicates (Hoang et al. 2018; Minh et al. 2020). Additionally, to further support the bootstrap values and assess the robustness of the inferred branches, the Shimodaira–Hasegawa‐like approximate likelihood ratio test (SH‐aLRT) was conducted with 1000 replicates (Guindon et al. 2010). The best‐fitting substitution models for nucleotide sequences were determined for each gene alignment using ‘ModelFinder’ in IQ‐TREE software with the ‘MFP + MERGE’ option, based on the Bayesian Information Criterion (BIC) (Kalyaanamoorthy et al. 2017). Data blocks for protein‐coding genes were pre‐defined to represent all three codon positions. The resulting tree was visualized using iTOL v5 (Letunic and Bork 2021) and further manually edited for clarity with Photoshop v.2023 (Adobe).

## 3 Results

### 3.1 Brief Surveys in Aowanda Forest and Tonglin Valley

In Aowanda National Forest Recreational Area, a total of 14 specimens of Culicidae and 8 specimens of Corethrellidae were collected using a trap equipped with a wireless speaker. In contrast, no specimens of either Culicidae or Corethrellidae were collected using the control trap without a wireless speaker. All collected mosquitoes, all of which were females, belonged to the genus *Uranotaenia*, known as ‘frog‐biting mosquitoes,’ except for one specimen of *Armigeres subalbatus*. In particular, *Ur*. *nivipleura* (13 specimens) was collected. All collected Corethrellidae are identified as *C. nippon* (Table 1). Other Diptera specimens included Cecidomyiidae, Mycetophilidae, Psychodidae, Phoridae, and Syrphidae, Sciaridae, with Cecidomyiidae being predominant. These taxa were also collected in the control group.

**TABLE 1:** Number of collected species by frog calling traps in Aowanda forest and Tonglin valley area.

Collected species	Aowanda forest (mountainous area)		Tonglin (valley area)	
	Mixed frog calls^a^	Control	Mixed frog calls	Control
Culicidae				
*Ur*. *nivipleura*	13	0	66	0
*Ur*. *macfarlanei*	0	0	20	0
*Mi*. *luzonensis*	0	0	5	0
*Ar. subalbatus*	1	0	0	0
Total	14	0	91	0
Corethrellidae				0
*C. Nippon*	8	0	1007	0
Total	8	0	1007	0

Abbreviations: Ar, *Armigeres*; C, *Corethrella*; Mi, *Mimomyia*; Ur, *Uranotaenia*.

^a^ Mixed frog call including the calls of *Hyla chinensis* , *Hylarana latouchii* , *Sylvirana guentheri* , *Micryletta steinegeri* , *Kurixalus eiffingeri* , *Fejervarya limnocharis* , *Zhangixalus moltrechti* , and *Odorrana swinhoana* . Control group did not play any frog call.

In the valley area of Tonglin, a total of 91 mosquito specimens were collected, representing the genera *Uranotaenia* and *Mimomyia*. Specifically, 66 of *Ur*. *nivipleura*, 20 of *Ur*. *macfarlanei*, and 5 of *Mimomyia luzonensis* (Ludlow, 1905) were collected from the two traps. Additionally, a total of 1007 Corethrellidae were collected, all of which were *C. nippon* (Table 1). No mosquitoes or frog‐biting midges were collected in the control group. However, other Diptera species were collected from both traps, including Sciaridae, Psychodidae, Drosophilidae, Chironomidae, Tipulidae, and Ceratopogonidae. Regarding mixed frog calls, a total of 46 Culicidae specimens (34 *Ur*. *nivipleura*, eight *Ur*. *macfarlanei*, and four of *Mi*. *luzonensis*) and 803 Corethrellidae (all *C. nippon* ) were collected in one night. In response to the calls of *M. steinegeri* , no mosquitoes or frog‐biting midges were collected. Conversely, the calls of *F. limnocharis* attracted eleven Culicidae specimens (nine *Ur*. *nivipleura*, two *Ur*. *macfarlanei*) and 58 Corethrellidae (all *C. nippon* ). The calls of *H. latouchii* attracted no Culicidae specimens and 17 Corethrellidae specimens. A total of five Culicidae (all *Ur*. *nivipleura*) and 39 Corethrellidae (all *C. nippon* ) specimens were collected in response to the calls of *Mi*. *fissipes*. In response to the calls of *Z. moltrechti* , 11 mosquitoes (10 *Ur. macfarlanei*, one *Ur. nivipleura*) and 75 Corethrellidae ( *C. nippon* ) were collected. The calls of *S. guentheri* did not attract any mosquitoes or frog‐biting midges. The calls of *H. chinensis* attracted no mosquitoes but two Corethrellidae ( *C. nippon* ). The calls of *R. sauteri* attracted eight mosquitoes (all *Ur. nivipleura*) and nine Corethrellidae ( *C. nippon* ). The calls of *O. swinhoana* attracted 16 mosquitoes (14 *Ur. nivipleura*, two *Ur. macfarlanei*) and 203 Corethrellidae ( *C. nippon* ). Lastly, the calls of *K. eiffingeri* attracted 14 mosquitoes (11 *Ur. nivipleura*, three *Ur. macfarlanei*) and 104 Corethrellidae ( *C. nippon* ). More detailed results are presented in Table 2. In addition, various families of Diptera, including Sciaridae, Psychodidae, Drosophilidae, Chironomidae, Tipulidae, and Ceratopogonidae, were collected from all types of frog calls without the use of lights, similar to those collected by the mixed frog call traps.

**TABLE 2:** Number of collected species by the mixed frog calls and each of the 4 species of frog call traps in the Tonglin valley area.

Collected species	Mixed frog calls	*M. steinegeri*	*F. limnocharis*	*H. latouchii*	*Mi*. *fissipes*	*Z. moltrechti*	*S. guentheri*	*Hy*. *chinensis*	*R. sauteri*	*O. swinhoana*	*K. eiffingeri*
Culicidae											
*Ur*. *nivipleura*	34	0	9	0	5	1	0	0	8	14	11
*Ur*. *macfarlanei*	8	0	2	0	0	10	0	0	0	2	3
*Mi*. *luzonensis*	4	0	0	0	0	0	0	0	0	0	0
Total	46	0	11	0	5	11	0	0	8	16	14
Corethrellidae											
*C. nippon*	803	0	58	17	39	75	0	2	9	203	104
Total	803	0	58	17	39	75	0	2	9	203	104

*Note:* Diptera genera abbreviations–Ar, *Armigeres*; C, *Corethrella*; Mi, *Mimomyia*; Ur, *Uranotaenia*. Frog genera abbreviations–F, *Fejervarya*; H, *Hylarana*; Hy, *Hyla*; K, *Kurixalus*; M, *Micryletta*; Mi, *Microhyla*; O, *Odorrana*; S, *Sylvirana*; R, *Rana*; Z, *Zhangixalus*.

### 3.2 DNA Barcodes and Mitochondrial Genomic Characteristics

In this study, partial mtCOI sequences were extracted for DNA barcodes from a total of five species, including *Ar*. *subalbatus*, *Ur*. *nivipleura*, *Ur*. *macfarlanei*, and *Mi*. *luzoensis* from the family Culicidae, as well as *C. nippon* from the family Corethrellidae. The corresponding GenBank accession numbers are as follows: *Ar*. *subalbatus*—PV576006, *Ur*. *nivipleura—*PV576008, *Ur*. *macfarlanei—*PV576009, *Mi*. *luzoensis—*PV576069, *C. nippon* —PV605830.

**FIGURE 6:**
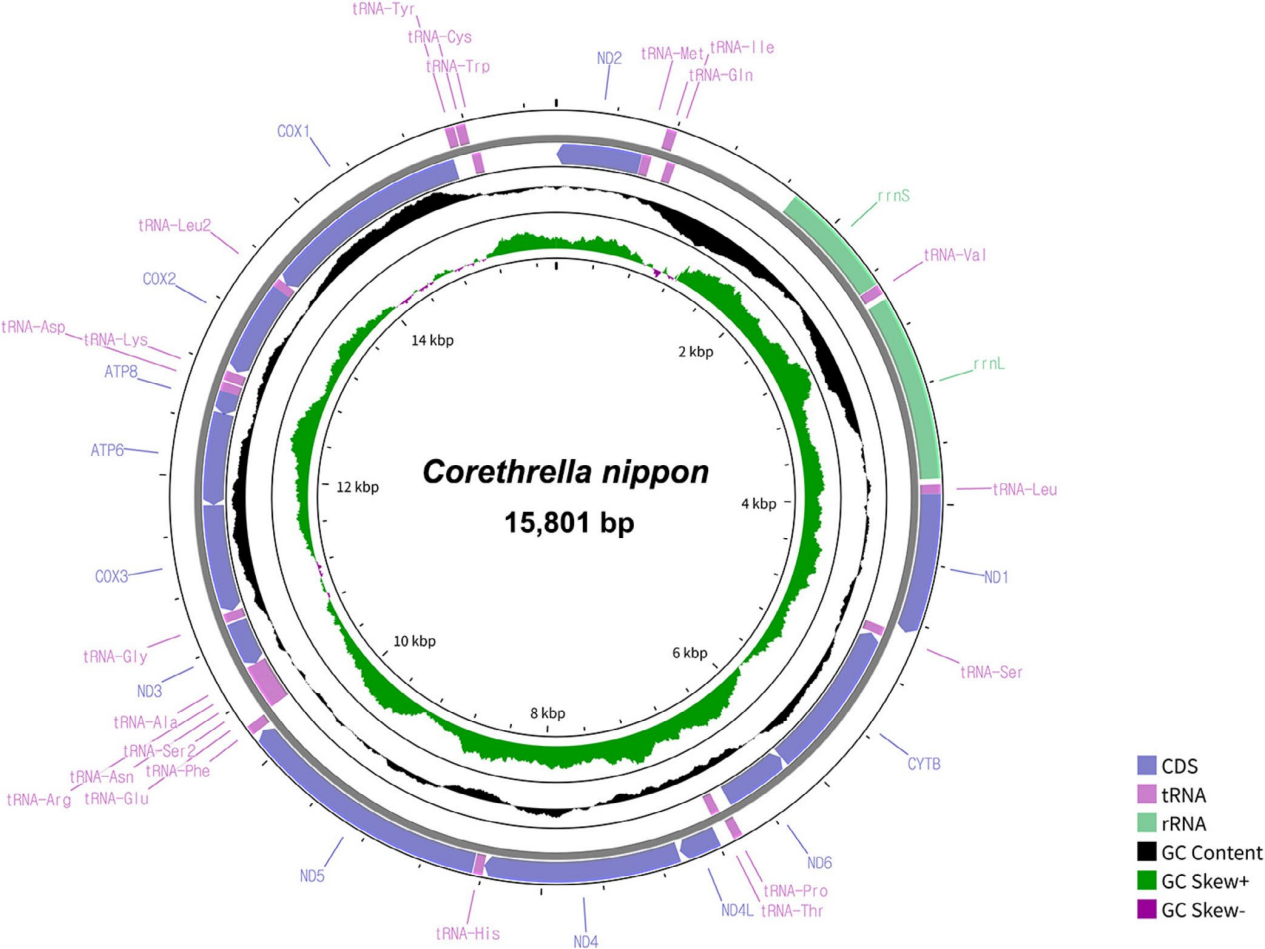
Mitochondrial genome circular structure of *Corethrella nippon* Miyagi 1980.

The extracted mitochondrial genome of *C. nippon* is 15,801 bp in length and is completely annotated, comprising 37 genes, including 22 transfer RNAs (tRNAs), 13 PCGs, and two ribosomal RNAs (rRNAs) (Figure 6). The base composition is as follows: A, 37.35%; T, 38.79%; G, 14.58%; and C, 9.29%, with a strong A + T bias (76.13%). The calculated GC skew was 0.22, indicating a higher proportion of guanine over cytosine, while the AT skew was −0.019, showing a slight excess of thymine over adenine. The PCGs predominantly used ATG as the start codon (COX3, CYTB, ATP6, ND4, and ND4L) or ATT (COX2, ND2, ND3, ND5, and ND6). Alternative start codons were also observed: TTG for ND1; TCG for COX1; ATC for ATP8. The stop codons exhibited variation as well, with TAA being the most common, utilized by COX1, COX3, CYTB, ND4, ND6, ATP6, and ATP8. This was followed by TAG, which was used by ND1, ND3, and ND4L. Additionally, incomplete stop codons (‘T—’) were present in COX2, ND2, and ND5, suggesting potential RNA editing or polyadenylation to complete the termination signal.

### 3.3 Mitogenome‐Based Phylogeny of Culicoidea Families

**FIGURE 7:**
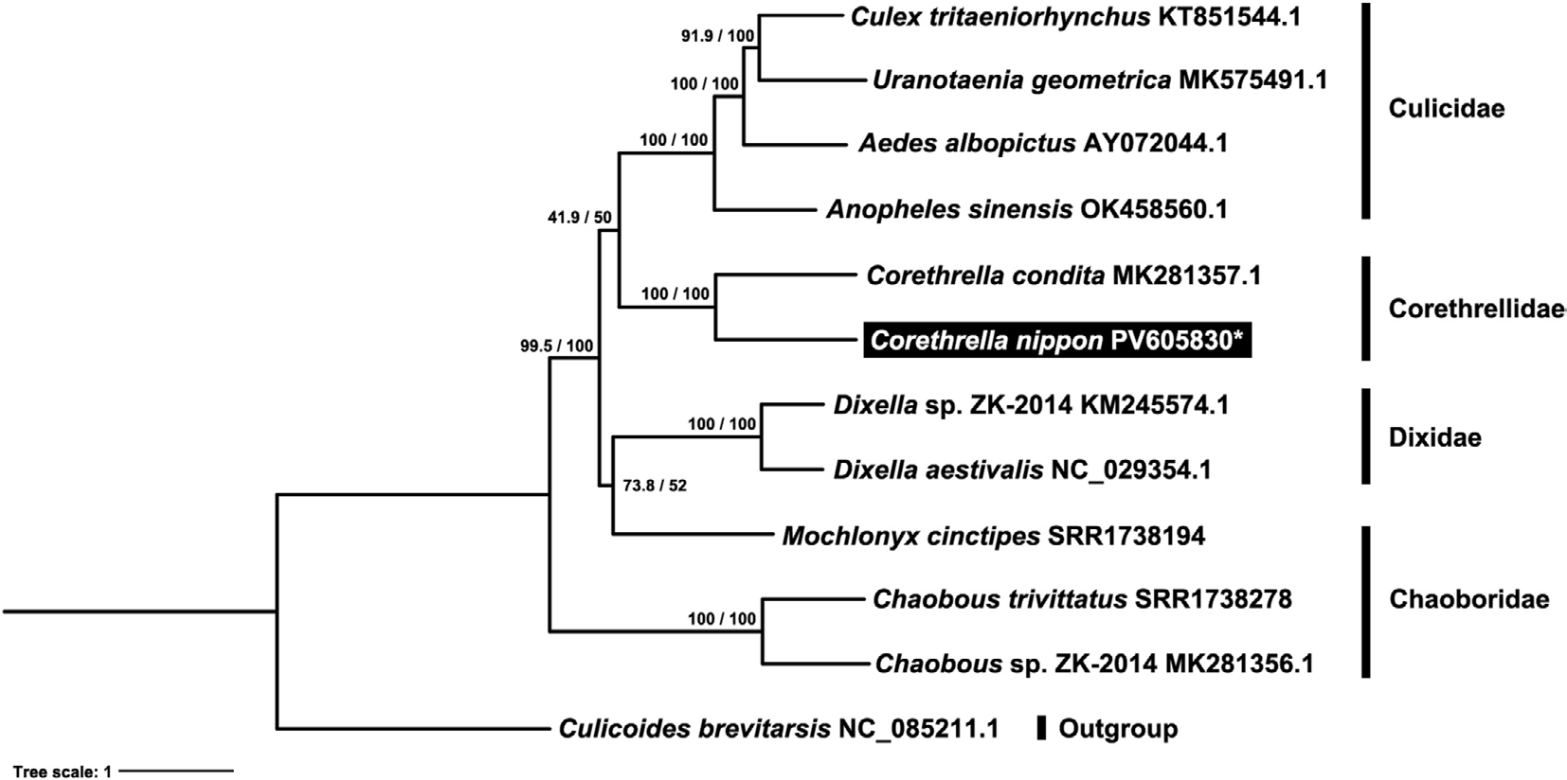
Maximum likelihood phylogeny of Culicoidea families using 13 PCGs of mitochondrial genomes, including four species of Culicidae, two species of Corethrellidae, two species of Dixidae, three species of Chaoboridae, and one outgroup as *Culicoides brevitarsis* of Ceratopogonidae. The white asterisk with a black rectangular box indicates the newly provided mitochondrial genome of *C. nippon* (accession: PV605830) in this study.

The phylogenetic analysis showed the monophyly of the families Culicidae and Corethrellidae. Within the family Chaoboridae, however, the genera *Mochlonyx* and *Chaoborus* formed distinct clades, indicating paraphyly within the family. Within the family Culicidae, genus *Anopheles* was resolved as the earliest‐diverging lineage, followed by the clades consisting of genera *Aedes* + (*Culex* + *Uranotaenia*). For the family Corethrellidae, the newly sequenced *Corethrella nippon* grouped with *C*. *condita* to form a well‐supported clade (SH‐aLRT = 100/bootstrap value = 100). This family was also placed phylogenetically closer to the family Culicidae (Figure 7).

## 4 Discussion

### 4.1 Newly Designed Traps

It is well known that hematophagous insects, such as mosquitoes or black flies, respond to carbon dioxide and odor when seeking hosts (Becker et al. 1995). However, contrary to the widely known fact, McKeever (1977) was the first to demonstrate that a large number of Corethrellidae could be collected in response to frog calls, independent of carbon dioxide or skin odor. This study also confirmed that a large number of Culicidae and Corethrellidae specimens were collected similarly.

First, we designed a new trap to collect and test the host preference by referencing the CDC‐miniature trap and those used in studies by McKeever (1977) and Toma et al. (2005). The trap features a 5 V CPU cooling fan and a wireless speaker equipped with an SD card slot, not using Bluetooth mode. It is powered by a 20,000 mAh power bank, allowing it to operate for approximately 40 h. Additionally, the trap is compact, easily detachable, and portable. The collection results showed a significant number of Culicidae and Corethrellidae specimens, comparable to those in Toma et al. (2005) and Toma et al. (2019), demonstrating the successful application of this trap in our study (Table 1).

### 4.2 The Result of Foraging Test Using the Trap

We collected the frog‐biting midges and mosquitoes using the calling sounds of local frog species in Taiwan. The mosquito species collected included *Ur*. *nivipleura*, *Ur*. *macfarlanei*, and *Mi*. *luzonensis*, with the highest number of specimens being *Ur*. *nivipleura*, followed by *Ur*. *macfarlanei* and *Mi*. *luzonensis*. It is widely known that mosquitoes of the genus *Uranotaenia* feed on amphibian blood, and this study also confirmed their attraction to native frog calls in Taiwan (Marks 1960; Remington 1945). Additionally, similar to the findings of Toma et al. (2005), the collection of *Mi*. *luzonensis* further demonstrated that mosquitoes of this genus are also attracted to amphibians (Toma et al. 2005, 2019; Van Beurden 1980). We experimented using different frog calls independently to see how many frog– *C. nippon* was collected. In this study, we cannot conclude that *C. nippon* has a specific preference. However, an interesting finding is that *C. nippon* was captured within the range of frog calls around 2300 Hz, and not many *C. nippon* were captured when using frog calls with frequencies higher than 3000 Hz. Previous studies have shown that the trapping efficiency of frog‐biting midges is negatively correlated with call frequency (Grafe et al. 2018), a trend consistent with the findings of this study. However, as noted by Virgo et al. (2022), acoustic signals are not the sole factor; other ecological traits such as host behavior, habitat characteristics, and skin properties may also play important roles in shaping these interactions. To disentangle these factors, it will be necessary to compare results obtained from experiments using live frogs in host preference studies with those from speaker‐based calling traps. We also observed frog species that called within the same 2000 Hz range but attracted relatively few midges, suggesting that pulsed sound structure, as emphasized by Meuche et al. (2016) and Virgo et al. (2019), warrants further investigation.

Future studies, we expect to conduct experiments on midge preference for frog calling using the new trap we have developed.

### 4.3 Implications of Molecular Data

The completely annotated mitochondrial genome of *C. nippon* was newly produced in this study. A partial mitogenome of *C*. *condita* has been previously registered in GenBank; however, this study represents the first complete annotation and registration of a mitogenome within the family Corethrellidae.

DNA barcodes of collected mosquito species are also presented, which are expected to serve as valuable references for species identification and future phylogenetic studies of frog‐biting dipteran species. Notably, the majority of molecular data for frog‐biting midges has been registered from Nearctic or Neotropical species, with this study providing the first molecular data from East Asia. In addition, this study contributes additional DNA barcode data for Taiwanese mosquitoes, expanding the study of Gao et al. (2017). In this study, we registered species from the genera *Uranotaenia* and *Mimomyia*, which predominantly feed on amphibians rather than act as vector‐borne species for humans.

### 4.4 Limitation of Phylogenetic Analysis of Culicoidea Families

The newly sequenced *Corethrella nippon* successfully grouped with the previously reported *C*. *condita*, forming a single clade. However, the phylogenetic relationships among the four families of Culicoidea in this study revealed several discrepancies compared to those identified in previous studies (Figure 7). Particularly, the family Chaoboridae was placed as the earliest‐diverging lineage within Culicoidea, in contrast to results from previous studies. Family Dixidae is commonly considered the earliest‐diverging family, while the position of families Corethrellidae and Chaoboridae relative to Culicidae remains controversial (Wiegmann et al. 2011; Borkent 2012). Previous mitogenome‐based phylogenies also revealed the uncertain placement of family Chaoboridae within Culicomorpha (Zhang et al. 2019; An et al. 2022). This raises the question of whether mitochondrial genome‐based molecular phylogenies at the multigene level are suitable for resolving phylogenetic relationships at the infraorder or superfamily level, especially when dealing with deep topologies (Hassanin et al. 2005; Burger et al. 2003; Lin and Danforth 2004; Talavera and Vila 2011). Furthermore, Kutty et al. (2018) showed the monophyly of four families within Culicoidea at the infraorder level. However, as their study was based on only 12 species of Culicoidea, more extensive research focusing on relationships at the family, subfamily, or genus levels within Culicoidea is needed.

The relationships between the two genera within Chaoboridae (genera *Mochlonyx* and *Chaoborus*) were found to be paraphyletic from our phylogenetic tree. In contrast, morphology‐based phylogenetic studies have consistently supported the monophyly of Chaoboridae (Ogawa 2007; Borkent 2012). This study represents the first molecular phylogenetic analysis of the relationship between these two genera. However, due to the limited dataset, these results are difficult to interpret with high confidence, highlighting the need to incorporate more molecular data from additional genera to better resolve the evolutionary relationships within the family.

For the family Dixidae, only a single genus, *Dixella*, currently has mitogenome data available in GenBank. Similar to Chaoboridae, future phylogenetic studies on this family should incorporate molecular data from additional genera. Since the phylogenetic relationships within Dixidae have been mostly investigated based on morphological traits, a comprehensive study of the family is necessary.

In family Culicidae, the results were consistent with previous studies, confirming that genus *Anopheles* is the earliest‐diverging lineage (Reidenbach et al. 2009; Soghigian et al. 2023; Chen et al. 2024). Additionally, families Culicidae and Corethrellidae were found to form a sister clade. This result contrasts with the transcriptome‐based phylogeny of Kutty et al. (2018) but aligns with the results of Wiegmann et al. (2011). Given the differences between these studies, further analyses of superfamily relationships are needed using more extensive datasets at the subfamily‐, tribe‐, and genus‐level scales.

Assuming that families Culicidae and Corethrellidae are indeed closely related, the results suggest that the evolution of blood‐feeding traits within Culicoidea may have originated from a common ancestor of the two families, implying that these traits did not evolve independently. Moreover, the specialized amphibian‐biting traits of Corethrellidae align with the comparative phylogenetic inference on host preference based on ancestral character reconstruction by Soghigian et al. (2023), which suggested that mosquitoes initially fed on amphibians. It showed the hypothesis that the common ancestor of Culicidae and Corethrellidae was also primarily an amphibian blood‐feeder.

However, the phylogenetic relationships within Culicoidea inferred in this study showed several limitations. The small number of taxa included, the generally low bootstrap values, and the inherent challenges of using mitochondrial genomes to resolve deep‐level topologies indicate the need for further research and more comprehensive datasets. Resolving these issues in future studies is expected to enable the reconstruction of not only the phylogenetic relationships among families but also the evolutionary trajectories of various traits.

Regarding Corethrellidae, Virgo et al. (2022) conducted a simple network analysis of host preference and frog species using two genetic markers: partial mtCOI and partial rRNA internal transcribed spacer 2 (ITS2). However, these markers alone provide limited resolution for inferring phylogenetic relationships and also for network analysis. Once sufficient data are accumulated—including both described and newly recorded species as well as their associations with frog hosts—more robust phylogenetic analyses within the family will become feasible, ultimately enabling phylogeny‐based interpretations of host preference and evolutionary patterns.

## 5 Conclusion

This study holds significant importance as it is the second study of Corethrellidae conducted in East Asia, following the research on Iriomote Island in the Ryukyu Archipelago, Japan, and the first in Taiwan by using the frog‐calling traps for investigation purposes. Additionally, we designed a new, advanced type of trap based on the CDC miniature trap and the trap used by McKeever (1977) and Toma et al. (2019), which successfully enabled the collection of a significant number of Culicidae and Corethrellidae specimens. As a result, the frog‐calling trap tended to collect a greater number of Culicidae and Corethrellidae in the valley area, with *O. swinhoana* and *K. eiffingeri* attracting the most specimens in that order. Through a comprehensive analysis of host preference, corethrellids were found to be most abundantly collected at a sound frequency of approximately 2022.2 Hz. In addition, the complete mitochondrial genome of the collected *C. nippon* was successfully extracted and completely annotated in this study. A preliminary phylogenetic analysis showed that *C. nippon* forms a sister clade with Culicidae, both of which have blood‐feeding habits. However, to infer a more robust deep topology at the superfamily level and estimate evolutionary histories, it is necessary to use other genomic datasets beyond the mitogenome and include additional taxa. In conclusion, this study serves as a foundation for further comprehensive research on Corethrellidae in East Asia and shows potential to advance our understanding of the ecological characteristics and evolutionary histories related to frog‐biting traits.

## Author Contributions

**Woo Jun Bang:** conceptualization (lead), data curation (equal), formal analysis (equal), investigation (lead), methodology (lead), project administration (lead), resources (equal), software (equal), supervision (lead), visualization (equal), writing – original draft (lead), writing – review and editing (lead). **Jh Yu You:** data curation (equal), formal analysis (lead), investigation (lead), methodology (equal), resources (equal), software (lead), validation (lead), writing – original draft (lead), writing – review and editing (lead). **Yoonhyuk Bae:** data curation (equal), formal analysis (equal), resources (equal), software (equal), validation (equal), writing – review and editing (equal). **Ming‐Feng Chuang:** investigation (equal), methodology (equal), project administration (equal), supervision (equal), validation (equal), writing – review and editing (equal). **Seunggwan Shin:** conceptualization (supporting), funding acquisition (lead), project administration (equal), supervision (equal), validation (equal), writing – review and editing (equal).

## Conflicts of Interest

The authors declare no conflicts of interest.

## Supporting information


**Figure S1:** Summary of newly designed trap components and assembly procedures (A–H).

## Data Availability

All data generated during this study are included in the article. Sequences used in this study are deposited in GenBank (https://www.ncbi.nlm.nih.gov/genbank/) as follows: PV576006, PV576008, PV576009, PV576069, PV605830.

## References

[ece372405-bib-0001] Allio, R. , A. Schomaker‐Bastos , J. Romiguier , F. Prosdocimi , B. Nabholz , and F. Delsuc . 2020. “MitoFinder: Efficient Automated Large‐Scale Extraction of Mitogenomic Data in Target Enrichment Phylogenomics.” Molecular Ecology Resources 20, no. 4: 892–905. 32243090 10.1111/1755-0998.13160 PMC7497042

[ece372405-bib-0002] An, Y. , C. Li , J. Li , and Y. Wang . 2022. “The Complete Mitochondrial Genome of *Simulium jisigouense* (Diptera: Simuliidae) and Phylogenetic Analysis of Simuliidae.” Frontiers in Ecology and Evolution 10: 932601.

[ece372405-bib-0003] Becker, N. , M. Zgomba , D. Petric , and M. Ludwig . 1995. “Comparison of Carbon Dioxide, Octenol and a Host‐Odour as Mosquito Attractants in the Upper Rhine Valley, Germany.” Medical and Veterinary Entomology 9, no. 4: 377–380. 8541587 10.1111/j.1365-2915.1995.tb00008.x

[ece372405-bib-0004] Bernal, X. E. , and P. de Silva . 2015. “Cues Used in Host‐Seeking Behavior by Frog‐Biting Midges (*Corethrella* spp. Coquillet).” Journal of Vector Ecology 40, no. 1: 122–128. 26047192 10.1111/jvec.12140

[ece372405-bib-0005] Bolger, A. M. , M. Lohse , and B. Usadel . 2014. “Trimmomatic: A Flexible Trimmer for Illumina Sequence Data.” Bioinformatics 30, no. 15: 2114–2120. 24695404 10.1093/bioinformatics/btu170 PMC4103590

[ece372405-bib-0006] Borkent, A. 2008. “The Frog‐Biting Midges of the World (*Corethrellidae*: Diptera).” Zootaxa 1804: 1–456.

[ece372405-bib-0007] Borkent, A. 2012. “The Pupae of the Biting Midges of the World (*Diptera*: Ceratopogonidae), With a Generic Key and Analysis of the Phylogenetic Relationships Between Genera.” Zootaxa 3879: 1–327. 10.11646/zootaxa.3879.1.1 25544570

[ece372405-bib-0008] Borkent, A. 2014. “World Catalog of Extant and Fossil Corethrellidae (*Diptera*).” Zootaxa 3796: 453–468. 10.11646/zootaxa.3796.3.3 24870687

[ece372405-bib-0009] Borkent, A. , and P. Belton . 2006. “Attraction of Female *Uranotaenia lowii* (*Diptera*: Culicidae) to Frog Calls in Costa Rica.” Canadian Entomologist 138: 91–94.

[ece372405-bib-0010] Burger, G. , M. W. Gray , and B. F. Lang . 2003. “Mitochondrial Genomes: Anything Goes.” Trends in Genetics 19, no. 12: 709–716. 14642752 10.1016/j.tig.2003.10.012

[ece372405-bib-0011] Chen, D. H. , S. L. He , W. B. Fu , et al. 2024. “Mitogenome‐Based Phylogeny of Mosquitoes (*Diptera*: Culicidae).” Insect Science 31, no. 2: 599–612. 37489338 10.1111/1744-7917.13251

[ece372405-bib-0012] Folmer, O. , M. Black , W. Hoeh , R. Lutz , and R. Vrijenhoek . 1994. “DNA Primers for Amplification of Mitochondrial Cytochrome c Oxidase Subunit I From Diverse Metazoan Invertebrates.” Molecular Marine Biology and Biotechnology 3, no. 5: 294–299. 7881515

[ece372405-bib-0013] Gao, B. , Y. Fang , J. Zhang , R. Wu , B. Xu , and L. Xie . 2017. “A DNA Barcoding Based Study to Identify Main Mosquito Species in Taiwan and Its Difference From Those in Mainland China.” Combinatorial Chemistry & High Throughput Screening 20, no. 2: 147–152. 28215143 10.2174/1386207320666170217153548

[ece372405-bib-0041] Grafe, T. U. , H. H. Ahmad Sah , N. Ahmad , A. Borkent , I. Meuche , and O. Konopik . 2018. “Studying the Sensory Ecology of Frog‐Biting Midges (Corethrellidae: Diptera) and Their Frog Hosts Using Ecological Interaction Networks.” Journal of Zoology 307, no. 1: 17–27.

[ece372405-bib-0014] Grant, J. R. , E. Enns , E. Marinier , et al. 2023. “Proksee: In‐Depth Characterization and Visualization of Bacterial Genomes.” Nucleic Acids Research 51, no. W1: W484–W492. 37140037 10.1093/nar/gkad326 PMC10320063

[ece372405-bib-0015] Guindon, S. , J. F. Dufayard , V. Lefort , M. Anisimova , W. Hordijk , and O. Gascuel . 2010. “New Algorithms and Methods to Estimate Maximum‐Likelihood Phylogenies: Assessing the Performance of PhyML 3.0.” Systematic Biology 59: 307–321. 20525638 10.1093/sysbio/syq010

[ece372405-bib-0016] Hassanin, A. , N. Léger , and J. Deutsch . 2005. “Evidence for Multiple Reversals of Asymmetric Mutational Constraints During the Evolution of the Mitochondrial Genome of Metazoa, and Consequences for Phylogenetic Inferences.” Systematic Biology 54, no. 2: 277–298. 16021696 10.1080/10635150590947843

[ece372405-bib-0017] Hoang, D. T. , O. Chernomor , A. von Haeseler , B. Q. Minh , and L. S. Vinh . 2018. “UFBoot2: Improving the Ultrafast Bootstrap Approximation.” Molecular Biology and Evolution 35: 518–522. 29077904 10.1093/molbev/msx281 PMC5850222

[ece372405-bib-0018] Kalyaanamoorthy, S. , B. Q. Minh , T. K. F. Wong , A. von Haeseler , and L. S. Jermiin . 2017. “ModelFinder: Fast Model Selection for Accurate Phylogenetic Estimates.” Nature Methods 14, no. 6: 587–589. 28481363 10.1038/nmeth.4285 PMC5453245

[ece372405-bib-0019] Kück, P. , and G. C. Longo . 2014. “FASconCAT‐G: Extensive Functions for Multiple Sequence Alignment Preparations Concerning Phylogenetic Studies.” Frontiers in Zoology 11: 81. 25426157 10.1186/s12983-014-0081-x PMC4243772

[ece372405-bib-0020] Kutty, S. N. , W. H. Wong , K. Meusemann , R. Meier , and P. S. Cranston . 2018. “A Phylogenomic Analysis of Culicomorpha (*Diptera*) Resolves the Relationships Among the Eight Constituent Families.” Systematic Entomology 43, no. 3: 434–446.

[ece372405-bib-0021] Letunic, I. , and P. Bork . 2021. “Interactive Tree of Life (iTOL) v5: An Online Tool for Phylogenetic Tree Display and Annotation.” Nucleic Acids Research 49: W293–W296. 33885785 10.1093/nar/gkab301 PMC8265157

[ece372405-bib-0022] Lien, J. C. 2004. “Pictorial Keys to the Mosquitoes of Taiwan: Key to the Species of Genus *Toxorhynchites* of Taiwan—Female Mosquitoes.” Yi Hsien.

[ece372405-bib-0023] Lien, J. C. , C. C. Lin , and M. H. Weng . 1998. “A New Record of *Corethrella nippon* Miyagi, 1980 From Taiwan (*Diptera*, *Chaoboridae*).” Chinese Journal of Parasitology 11: 91–97.

[ece372405-bib-0024] Lin, C. P. , and B. N. Danforth . 2004. “How Do Insect Nuclear and Mitochondrial Gene Substitution Patterns Differ? Insights From Bayesian Analyses of Combined Datasets.” Molecular Phylogenetics and Evolution 30, no. 3: 686–702. 15012948 10.1016/S1055-7903(03)00241-0

[ece372405-bib-0038] Marks, E. 1960. “Mosquito Biting Frogs.” Australian Journal of Science 23: 89.

[ece372405-bib-0025] McKeever, S. 1977. “Observations of *Corethrella* Feeding on Tree Frogs (*Hyla*).” Mosquito News 37: 522–523.

[ece372405-bib-0026] Meuche, I. , A. Keller , H. H. Ahmad Sah , N. Ahmad , and T. U. Grafe . 2016. “Silent Listeners: Can Preferences of Eavesdropping Midges Predict Their Hosts' Parasitism Risk?” Behavioral Ecology 27, no. 4: 995–1003.

[ece372405-bib-0027] Minh, B. Q. , H. A. Schmidt , O. Chernomor , et al. 2020. “IQ‐TREE 2: New Models and Efficient Methods for Phylogenetic Inference in the Genomic Era.” Molecular Biology and Evolution 37: 1530–1534. 32011700 10.1093/molbev/msaa015 PMC7182206

[ece372405-bib-0037] Miyagi, I. 1980. “Notes on the Japanese Species of the Genus Corethrella with Description of a New Species (Diptera, Chaoboridae).” Japanese Journal of Sanitary Zoology 31, no. 1: 15–21.

[ece372405-bib-0044] Ogawa, J. R. 2007. Phylogeny of the “Chaoboriform” Genera (Doctoral dissertation), 256. Oregon State University.

[ece372405-bib-0028] Reidenbach, K. R. , S. Cook , M. A. Bertone , R. E. Harbach , B. M. Wiegmann , and N. J. Besansky . 2009. “Phylogenetic Analysis and Temporal Diversification of Mosquitoes (*Diptera*: Culicidae) Based on Nuclear Genes and Morphology.” BMC Evolutionary Biology 9: 298. 20028549 10.1186/1471-2148-9-298 PMC2805638

[ece372405-bib-0039] Remington, C. L. 1945. “The Feeding Habits of Urano · taenia 10ωii Theobald (Diptera: Culicidae).” Entomological News 56: 32–68.

[ece372405-bib-0029] Soghigian, J. , C. Sither , S. A. Justi , et al. 2023. “Phylogenomics Reveals the History of Host Use in Mosquitoes.” Nature Communications 14, no. 1: 6252. 10.1038/s41467-023-41764-y PMC10558525 37803007

[ece372405-bib-0034] Stone, A. 1968. “The Genus Corethrella in the United States (Diptera: Chaoboridae).” Florida Entomologist 51, no. 3: 183–186. 10.2307/3493552.

[ece372405-bib-0043] Talavera, G. , and R. Vila . 2011. “What Is the Phylogenetic Signal Limit from Mitogenomes? The Reconciliation Between Mitochondrial and Nuclear Data in the Insecta Class Phylogeny.” BMC Evolutionary Biology 11, no. 1: 315. 22032248 10.1186/1471-2148-11-315 PMC3213125

[ece372405-bib-0035] Toma, T. , I. Miyagi , Y. Higa , T. Okazawa , and H. Sasaki . 2005. “Culicid and Chaoborid Flies (Diptera : Culicidae and Chaoboridae) Attracted to a CDC Miniature Frog Call Trap at Iriomote Island, the Ryukyu Archipelago, Japan.” Medical Entomology and Zoology 56, no. 2: 65–71.

[ece372405-bib-0036] Toma, T. , T. Takara , I. Miyagi , K. Futami , and Y. Higa . 2019. “Mosquitoes and Frog‐Biting Midges (Diptera: Culicidae and Corethrellidae) Attracted to Traps with Natural Frog Calls and Synthesized Sounds at Iriomote Island, Ryukyu Archipelago, Japan.” Medical Entomology and Zoology 70, no. 4: 221–234.

[ece372405-bib-0040] Van Beurden, E. K. 1980. “Mosquitoes (*Mimomyia elegans* (Taylor)) Feeding on the Introduced Toad *Bufo marinus* (Linnaeus): Implications for Control of a Toad Pest.” Australian Zoologist 20: 501–504.

[ece372405-bib-0030] Virgo, J. , A. Ruppert , K. P. Lampert , T. U. Grafe , and T. Eltz . 2019. “The Sound of a Blood Meal: Acoustic Ecology of Frog‐Biting Midges (*Corethrella*) in Lowland Pacific Costa Rica.” Ethology 125, no. 5: 354–361.

[ece372405-bib-0031] Virgo, J. , A. Ruppert , K. P. Lampert , T. U. Grafe , and T. Eltz . 2022. “More Than Meets the Eye: Decrypting Diversity Reveals Hidden Interaction Specificity Between Frogs and Frog‐Biting Midges.” Ecological Entomology 47, no. 1: 95–108.

[ece372405-bib-0042] Wiegmann, B. M. , M. D. Trautwein , I. S. Winkler , et al. 2011. “Episodic Radiations in the Fly Tree of Life.” Proceedings of the National Academy of Sciences 108, no. 14: 5690–5695. 10.1073/pnas.1012675108 PMC3078341 21402926

[ece372405-bib-0032] Zhang, X. , Z. Kang , S. Ding , Y. Wang , C. Borkent , and T. Saigusa . 2019. “Mitochondrial Genomes Provide Insights Into the Phylogeny of Culicomorpha (*Insecta*: *Diptera*).” International Journal of Molecular Sciences 20, no. 3: 747. 30754618 10.3390/ijms20030747 PMC6387087

[ece372405-bib-0033] Zhao, L. , J. Wang , H. Zhang , et al. 2022. “Parasite Defensive Limb Movements Enhance Acoustic Signal Attraction in Male Little Torrent Frogs.” eLife 11: e76083. 35522043 10.7554/eLife.76083 PMC9122496

