# Supplementary material for: Preliminary Study on Host Use and Phylogenetic Analysis of Corethrella nippon in Taiwan
Source: Ecol Evol. 2025 Oct 30;15(11):e72405. doi: 10.1002/ece3.72405 (PMC12572733; doi:10.1002/ece3.72405)
Supplement: Supplementary file 1 — Figure S1: Summary of newly designed trap components and assembly procedures (A–H). [file ECE3-15-e72405-s001.docx]

**
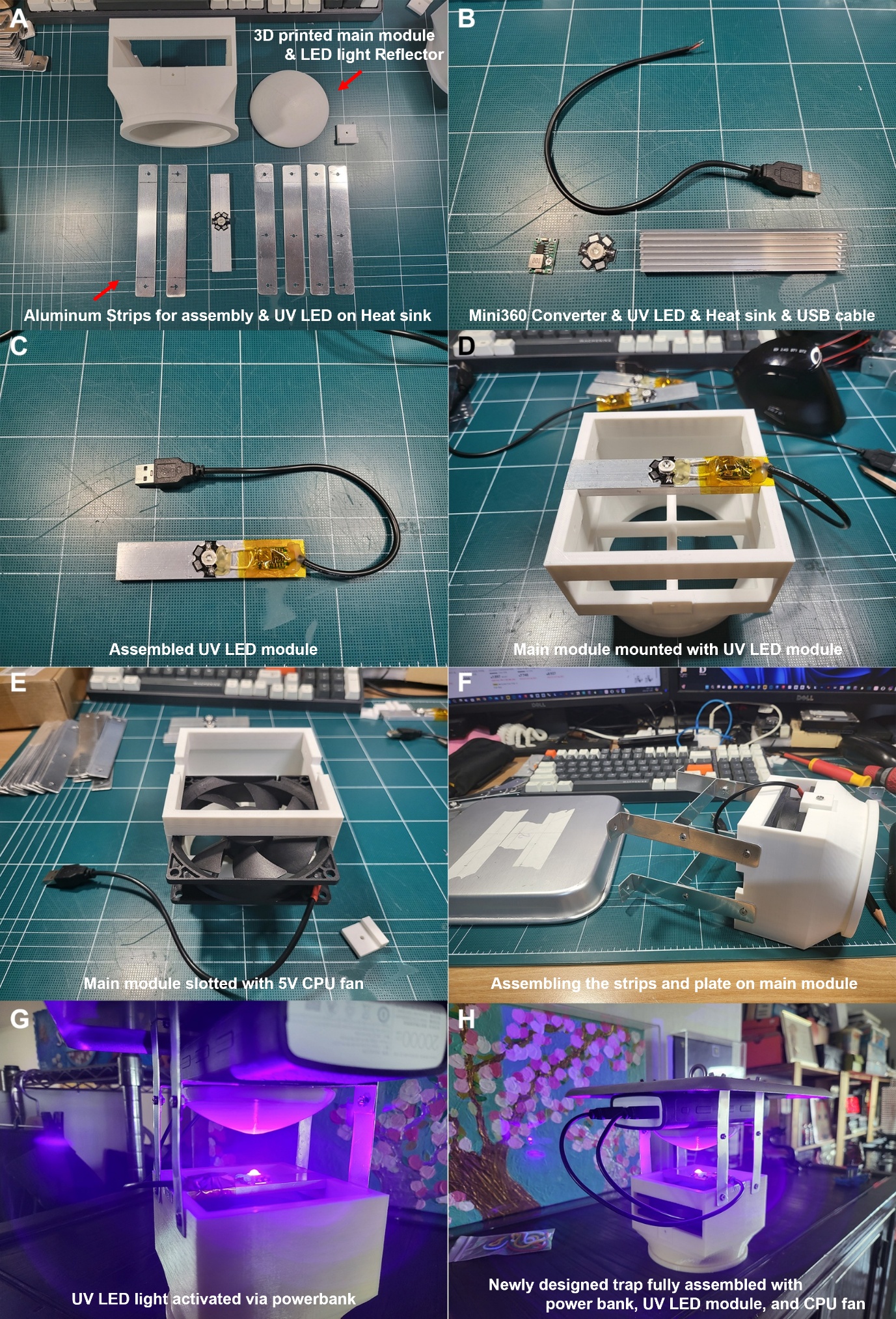
Figure S1**

**Figure legend**

**Figure S1.** Summary of newly designed trap components and assembly procedures (A–H)
